# Evaluating biomarkers in canine cytotoxic interface dermatitis reactions to account for clinical and histopathological similarities and differences

**DOI:** 10.3389/fvets.2024.1471590

**Published:** 2025-01-22

**Authors:** Shriya Kannan, Neil B. Wong, Grace E. Ryan, Nia E. R. James, Ayodeji Ajayi, Janet E. Lubov, Clement N. David, Linda Wrijil, Nicholas A. Robinson, Kelly Hughes, Ramon M. Almela, Jillian M. Richmond

**Affiliations:** ^1^Department of Dermatology, UMass Chan Medical School, Worcester, MA, United States; ^2^NanoString Technologies, Seattle, WA, United States; ^3^Pathology Department, Tufts Cummings School of Veterinary Medicine, North Grafton, MA, United States; ^4^Pathology Department, Colorado State University, Fort Collins, CO, United States; ^5^Department of Clinical Sciences, Tufts Cummings School of Veterinary Medicine, North Grafton, MA, United States

**Keywords:** cytotoxic interface dermatitis (CID), discoid lupus erythematosus (DLE), epitheliotropic lymphoma (EL)/cutaneous T cell lymphoma (CTCL), pemphigus, erythema multiforme, Vogt–Koyanagi–Harada (VKH), canine (dog), pharmaco-transcriptomics

## Abstract

Cytotoxic interface dermatitis (CID) is a pattern reaction predominantly at the dermo-epidermal junction that encapsulates numerous chronic non-communicable inflammatory skin conditions in which the basal keratinocytes are attacked by T-cell infiltrate leading to apoptosis, lymphocytic satellitosis and vacuolar degeneration. Though many diseases include CID, the type of clinical presentation and tissue patterns expressed from disease to disease varies. In this study, we evaluate the commonalities and discrepancies in significantly expressed biomarkers across several CID conditions to examine their impact on clinical presentations in canines. Among the uniquely expressed genes in each disease, we observed significantly expressed *IFNG* in Discoid Lupus Erythematosus, *TRAT1* in Epitheliotropic Lymphoma, and *CXCL8* and *CSF3R* in pemphigus affected dogs. With this knowledge, we may be able to use molecular signatures in combination with current treatment practices to develop a more targeted treatment plan for patients with CID.

## Introduction

Cytotoxic interface dermatitis (CID) is a cutaneous immunological reaction at the dermal-epidermal junction (DEJ) (1). The mononuclear cell-rich infiltrate (usually lymphocytic) may obscure the DEJ (2). However, the hallmark to this pattern is keratinocyte cell death confirmed by the presence of apoptotic keratinocytes, evidence of vacuolar degeneration at the DEJ, and satellitosis. CID is observed in different autoimmune and immune-mediated conditions such as erythema multiforme (EM) and variants of Cutaneous Lupus Erythematosus (CLE) (3). These conditions are notoriously difficult to distinguish histologically and often require trial and error to achieve treatment responses.

Clinical management of CID, however, remains unsatisfactory. Due to the difficulty associated with diagnosing and managing these cutaneous conditions and overlap syndromes, many medications are limited in their effectiveness in a patient. Topical immunosuppressants and products such as corticosteroids are currently being prescribed to manage disorders such as DLE in both humans and companion animals (4, 5). For more severe cases of cutaneous disorders such as EM, global immunosuppressive therapy is required which poses significant side effects (6–8).

Pet dogs develop CID conditions just like their human companions. Dogs may serve as an important model for CID given that they share a similar pathomechanism, molecular signature, environment and sometimes diet as people they live with. In this paper, we sought to discover biomarkers and cell types for a variety of CID conditions in dogs to identify molecular signatures for diagnosis and potentially targeted medications that could extend to similar disorders. We present data demonstrating shared and unique differentially expressed genes (DEGs) with protein-level confirmation using immunohistochemistry.

## Results

### Gene expression analysis in cytotoxic interface dermatitis reveals clusters by clinical subtypes

**Figure 1:**
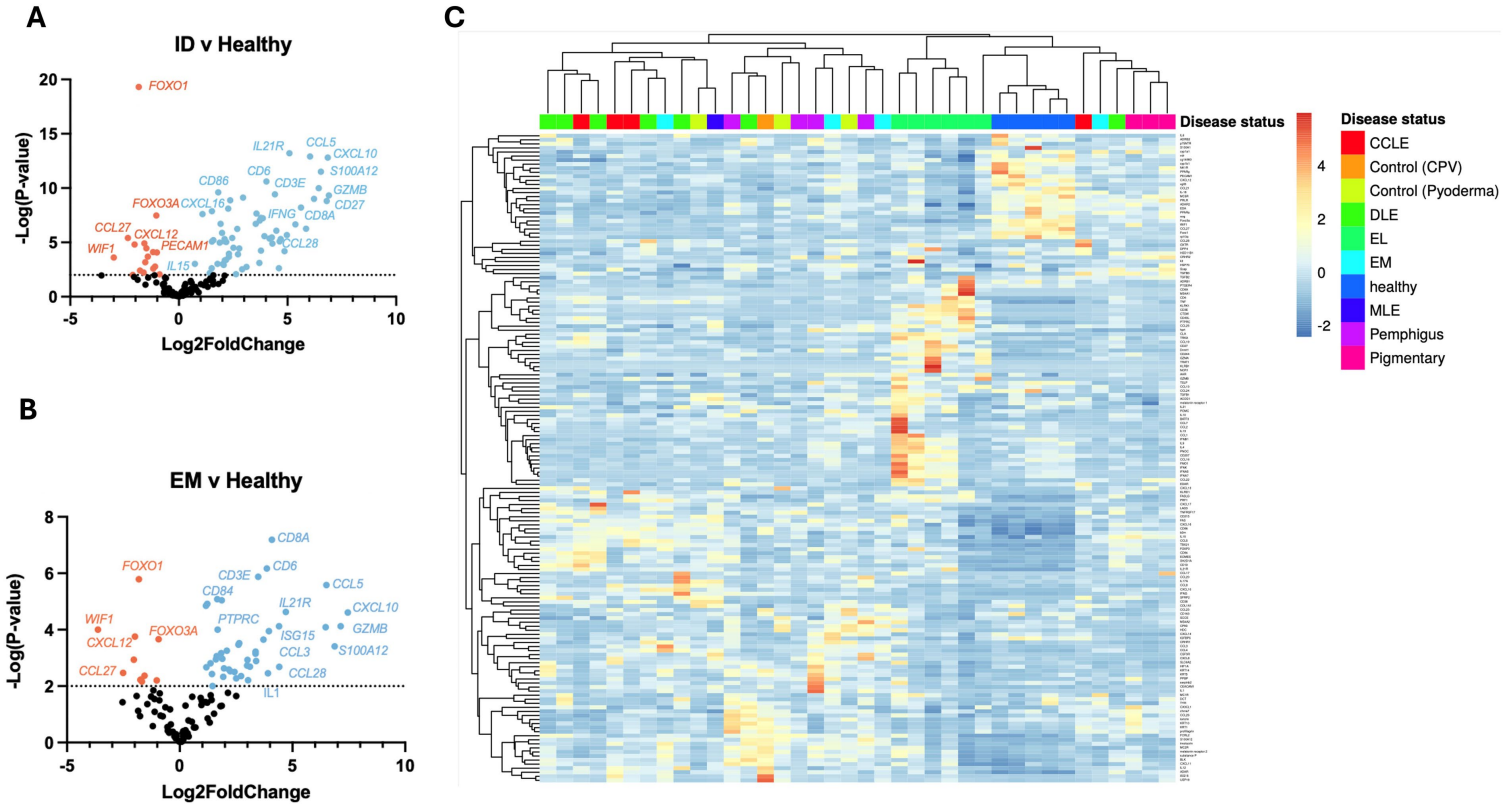
Examining gene expression in a discovery cohort of different clinical forms of canine CID reveals both unique and shared gene signatures. **(A)** Volcano plot of all CID samples versus healthy skin. **(B)** Volcano plot of a canine erythema multiforme (EM) dataset. **(C)** Heatmap with agglomerative clustering of all canine CID cases in the discovery cohort compared to healthy skin (*n* = 4 CCLE, 6 DLE, 6 EL, 4 EM, 4 pemphigus, 3 pigmentary and 5 healthy skin samples analyzed with a custom 160 gene probeset).

Using the NanoString nCounter platform, we performed a meta-analysis of our previously published case series, and an unbiased gene expression analysis in a discovery cohort of erythema multiforme (EM) samples from the Tufts biorepository. In total, these cases included 4 chronic cutaneous lupus erythematosus (9), 7 discoid lupus erythematosus (DLE) (10), 6 epitheliotropic lymphoma (EL) (11), 4 pemphigus variants (12) and 3 pigmentary disorders including 1 vitiligo and 2 Vogt–Koyanagi–Harada (VKH)-like cases (13) (detailed clinical presentation in Table 1). Here, we present 4 erythema multiforme (EM) cases, 3 pyoderma cases, 1 cell poor vasculitis (CPV) case, and 1 mucocutaneous lupus erythematosus (MLE) case. We compared true CID to other inflammatory conditions that do not attack the basal keratinocytes like pyoderma and CPV as well as to healthy skin controls that were obtained from leg amputations. We found 79 differentially expressed genes (DEGs) with a log_2_FC > 1.5 (56 up, 13 down) among DLE samples, 45 significant DEGs (34 up, 11 down) among EL samples, 51 significant DEGs (40 up, 11 down) among EM samples, 56 significant DEGs (44 up, 12 down) among Pemphigus samples, and 51 significant DEGs (42 up, 9 down) among Pigmentary disorder samples (Figures 1A,B). Next, to find a relationship between different CID cases we performed hierarchical clustering to organize disorders in the 38 dogs based on gene expression status (Figure 1C). We found that many cases clustered together, with a few cases exhibiting gene expression overlap across conditions.

**Table 1:** Discovery cohort signalments.

Case and diagnosis	GSM #	Signalment	Breed
CCLE, case 1	GSM5457071	7 yo, MN	German Shepherd
CCLE, case 2	GSM5457072	17 yo, MN	Mixed
CCLE, case 3	GSM5457073	5 yo, MN	Miniature Pinscher
CCLE, case 4	GSM5457074	6 yo, FS	West Highland White Terrier
DLE, case 1	GSM4869940	7 yo, MN	German Shepherd Cross
DLE, case 2	GSM4869942	9 yo, FS	Pitbull
DLE, case 3	GSM4869943	1 yo, FS	Boxer
DLE, case 4	GSM4869944	13 yo, MN	German Short Hair Pointer
DLE, case 5	GSM4869945	3 yo, MN	Saint Bernard
DLE, case 6	GSM4869946	5 yo, M	Mixed
DLE, case 7	GSM4869947	6 yo, MN	Coonhound
EL, case 1	GSM6506809	11 yo, FS	Labrador Retriever
EL, case 2	GSM6506810	13 yo, MN	Labrador Cross
EL, case 3	GSM6506811	9 yo, FS	Bloodhound
EL, case 4	GSM6506812	11 yo, MN	Dachshund
EL, case 5	GSM6506813	13 yo, MN	Golden Retriever
EL, case 6	GSM6506814	11 yo, FS	Olde English Bulldogge
EM, case 1	GSM7165607	11 yo, FS	Labrador Retriever
EM, case 2	GSM8302817	5 yo, FS	Boxer
EM, case 3	GSM7165608	11 yo, MN	Pomeranian
EM, case 4	GSM7165609	3 yo, MN	Shih Tzu
MLE, case 1	GSM4869948	6 yo, FS	Australian Shepherd
Pemphigus, case 1	GSM5218412	9 yo, FS	Labrador Cross
Pemphigus, case 2	GSM5218413	7 yo, FS	German Shepherd Cross
Pemphigus, case 3	GSM5218411	10 yo, FS	Miniature Schnauzer
Pemphigus, case 4	GSM5237149	12 yo, MN	Portuguese Water Dog
VKH, case 1	GSM4661969	2.5 yo, M	Bernese Mountain Dog
VKH, case 2	GSM4661970	2 yo, M	Husky
Vitiligo, case 1	GSM4661971	1 yo, MN	Labrador Cross

Comparatives and Diagnosis	GSM #	Signalment	Breed
Control (CPV), case 1	GSM8302818	4 yo, FS	Pomeranian
Control (Pyoderma), case 1	GSM8302820	7 yo, MN	Golden Retriever
Control (Pyoderma), case 2	GSM8302819	9 yo, MN	German Shepherd
Control (Pyoderma), case 3	GSM4869949	9 yo, M	Border Collie
Healthy, case 1	GSM4661972	6 yo, FS	Alaskan Malamute
Healthy, case 2	GSM4661973	12 yo, MN	German Shepherd Cross
Healthy, case 3	GSM4661974	8 yo, FS	Labrador Retriever
Healthy, case 4	GSM4661975	11 yo, MN	Golden Retriever
Healthy, case 5	GSM4661976	11 yo, FS	Siberian Husky Cross

FS, female spayed; MN, male neutered.

**Figure 2:**
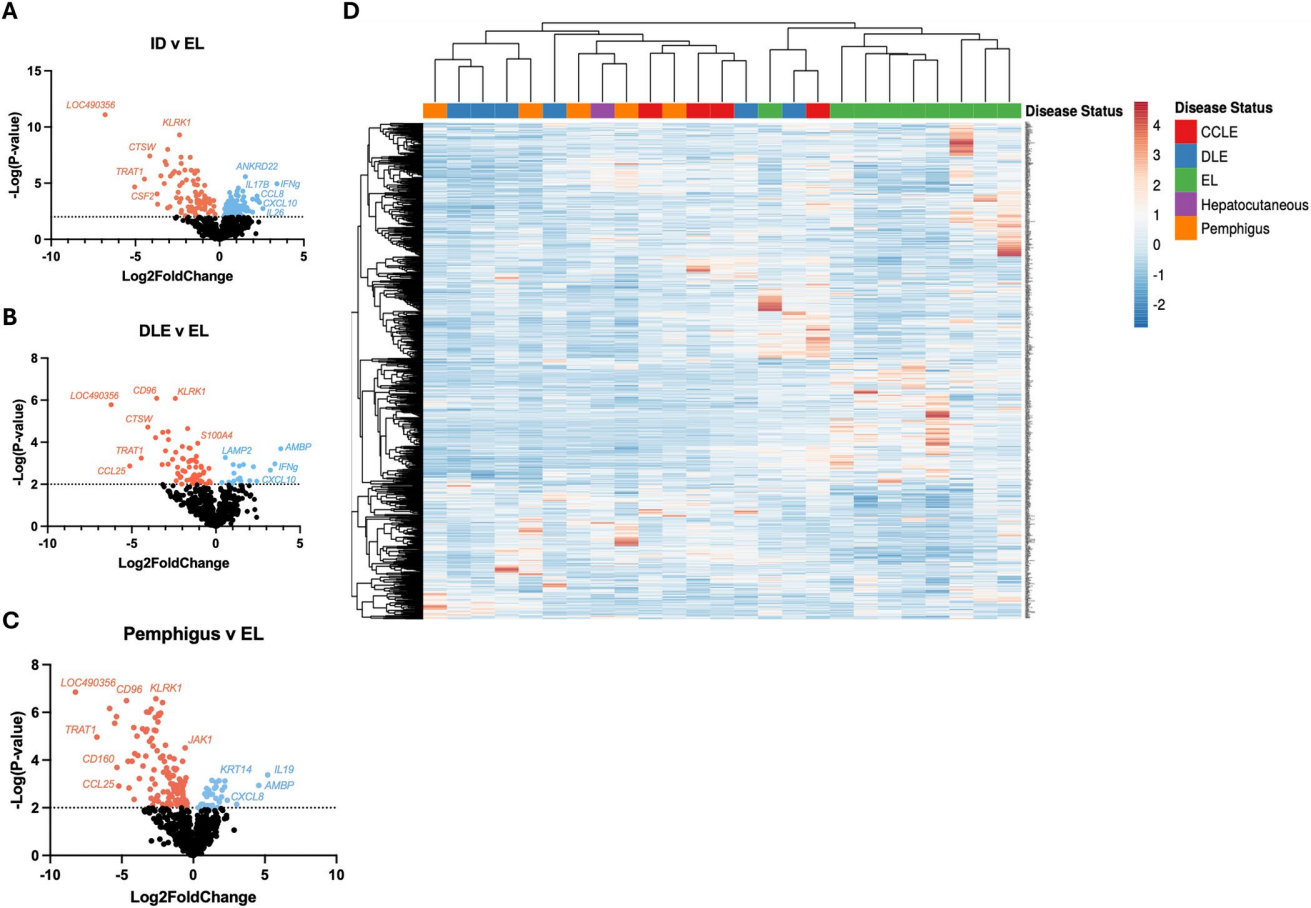
Examining gene expression in a validation cohort of different clinical forms of canine CID reinforces common and unique gene expression signatures. **(A)** Volcano plot of all CID samples versus epithelial lymphoma (EL) samples. **(B)** Volcano plot of a discoid lupus erythematosus (DLE) dataset. **(C)** Volcano plot of a pemphigus dataset. **(D)** Heatmap with agglomerative clustering of all canine CID cases in the validation cohort (*n* = 4 CCLE, 6 DLE, 9 EL, 1 HCS, 5 pemphigus, analyzed with a custom 800 gene probeset).

We then analyzed a validation cohort of 25 dogs (Table 2). Using the NanoString nCounter platform, we compared inflammatory skin disease samples to samples with EL exhibiting 167 significant DEGs (Figure 2A). We also compared DLE and Pemphigus samples to EL margins exhibiting 86 and 145 DEGs, respectively (Figures 2B,C) Using hierarchical clustering we again organized many disorders based on disease status (Figure 2D).

**Table 2:** Validation cohort signalments.

Case and Diagnosis	GSM #	Signalment	Breed
CCLE, case 1	GSM5457071	7 yo, MN	German Shepherd
CCLE, case 2	GSM5457072	17 yo, MN	Mixed
CCLE, case 3	GSM5457073	5 yo, MN	Miniature Pinscher
CCLE, case 4	GSM5457074	6 yo, FS	West Highland White Terrier
DLE, case 1	GSM4869940	7 yo, MN	German Shepherd Cross
DLE, case 2	GSM4869942	9 yo, FS	Pitbull
DLE, case 3	GSM4869943	1 yo, FS	Boxer
DLE, case 4	GSM4869944	13 yo, MN	German Short Hair Pointer
DLE, case 5	GSM4869945	3 yo, MN	Saint Bernard
DLE, case 6	GSM4869946	5 yo, M	Mixed
DLE, case 7	GSM4869947	6 yo, MN	Coonhound
EL, case 1	GSM6506809	11 yo, FS	Labrador Retriever
EL, case 2	GSM6506810	13 yo, MN	Labrador Cross
EL, case 3	GSM6506811	9 yo, FS	Bloodhound
EL, case 4	GSM6506812	11 yo, MN	Dachshund
EL, case 5	GSM6506813	13 yo, MN	Golden Retriever
EL, case 6	GSM6506814	11 yo, FS	Olde English Bulldogge
EM, case 1	GSM7165607	11 yo, FS	Labrador Retriever
EM, case 2	GSM8302817	5 yo, FS	Boxer
EM, case 3	GSM7165608	11 yo, MN	Pomeranian
EM, case 4	GSM7165609	3 yo, MN	Shih Tzu
MLE, case 1	GSM4869948	6 yo, FS	Australian Shepherd
Pemphigus, case 1	GSM5218412	9 yo, FS	Labrador Cross
Pemphigus, case 2	GSM5218413	7 yo, FS	German Shepherd Cross
Pemphigus, case 3	GSM5218411	10 yo, FS	Miniature Schnauzer
Pemphigus, case 4	GSM5237149	12 yo, MN	Portuguese Water Dog
VKH, case 1	GSM4661969	2.5 yo, M	Bernese Mountain Dog
VKH, case 2	GSM4661970	2 yo, M	Husky
Vitiligo, case 1	GSM4661971	1 yo, MN	Labrador Cross

Comparatives and diagnosis	GSM #	Signalment	Breed
Control (CPV), case 1	GSM8302818	4 yo, FS	Pomeranian
Control (Pyoderma), case 1	GSM8302820	7 yo, MN	Golden Retriever
Control (Pyoderma), case 2	GSM8302819	9 yo, MN	German Shepherd
Control (Pyoderma), case 3	GSM4869949	9 yo, M	Border Collie
Healthy, case 1	GSM4661972	6 yo, FS	Alaskan Malamute
Healthy, case 2	GSM4661973	12 yo, MN	German Shepherd Cross
Healthy, case 3	GSM4661974	8 yo, FS	Labrador Retriever
Healthy, case 4	GSM4661975	11 yo, MN	Golden Retriever
Healthy, case 5	GSM4661976	11 yo, FS	Siberian Husky Cross

FS, female spayed; MN, male neutered.

### Examination of differentially expressed genes reveals unique biomarkers of specific skin conditions

**Figure 3:**
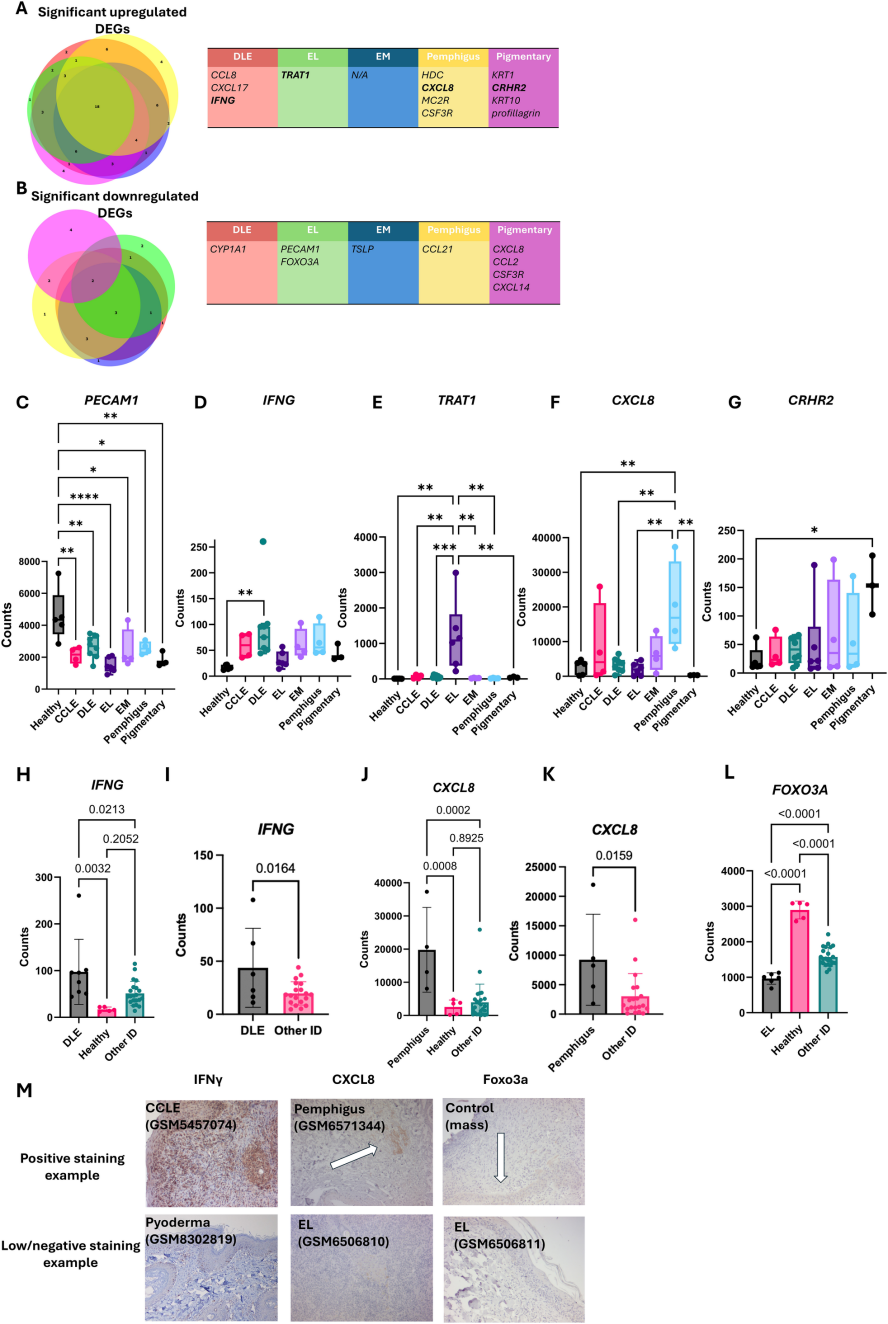
Examining unique gene expression in different clinical forms of canine CID reveals potential biomarkers of disease. **(A)** BioVenn diagrams demonstrating the overlap between gene signatures and the discrepancies that were upregulated in CID disorders. **(B)** BioVenn diagrams demonstrating the overlap between gene signatures and the discrepancies that were downregulated in CID disorders. Genes unique to specific disease entities in the discovery cohort (one-way ANOVAs with Tukey’s posttests significant as indicated) included **(C)** *PECAM1* in healthy skin, **(D)** *IFNG* in DLE, **(E)** *TRAT1* in EL, **(F)** *CXCL8* in pemphigus, and **(G)** *CRHR2* in pigmentary disorders. Examination of uniquely expressed genes in the **(H)** discovery and **(I)** validation cohorts of dogs for *IFNG*, **(J)** discovery and **(K)** validation for *CXCL8*, and **(L)** *FOXO3A* gene expression in the discovery cohort. *FOXO3A* was not included in the gene panel used for the validation cohort. **(M)** Example histology staining using anti-canine IFN-*γ*, CXCL8 and Foxo3A. Examples indicate positive IFN-γ staining in DLE, CXCL8 in pemphigus and a loss of FOXO3A staining in EL compared to a mass staining control, matching the gene expression analyses (*n* = 4 CCLE, 6 DLE, 6 EL, 4 EM, 4 pemphigus, 3 pigmentary and 5 healthy skin samples analyzed with a custom 160 gene probeset) (*n* = 4 CCLE, 6 DLE, 9 EL, 1 HCS, 5 pemphigus, analyzed with a custom 800 gene probeset).

Using BioVenn (14) to organize the upregulated and downregulated genes, we were able to discover overlapping and unique gene signatures across the different types of CID. Specifically, we noted a unique expression of *IFNG* in DLE (*p* = 0.0213), a unique expression of *TRAT1* and *FOXO3A* in EL patients (*p* < 0.0001 and *p* < 0.0001), and *CXCL8* and *CSF3R* in pemphigus patients (*p* = 0.0002 and *p* = 0.0016) (Figures 3A,B). We then compared the gene expression of unique genes per disease type against healthy skin and other CID samples in the discovery cohort. We noted that healthy leg skin uniquely expressed *PECAM1* (Figure 3C). *IFNG* was significantly upregulated in DLE (*p* = 0.0237) and trending toward significance in other CID conditions (Figure 3D). *TRAT1* was significantly elevated in EL (*p* = 0.013, Figure 3E). *CXCL8* was significantly upregulated in Pemphigus (*p* = 0.0053, Figure 3F). *CRHR2* was significantly elevated in pigmentary disorders (*p* = 0.0353, Figure 3G).

We further examined the DEGs identified in Figure 3 by comparing each condition to pooled other conditions in both the discovery and validation cohorts. We validated significant upregulation of *IFNG* in DLE (*p* ~ 0.01) (Figures 3H,I), and a significant upregulation of *CXCL8* and *CSF3R* in pemphigus (Figures 3J,K and Supplementary Figure S1). We also noted a significant downregulation of *FOXO3A*, a regulatory gene for apoptosis, within EL patients (Figure 3L, *P* < 0.0001). This gene was not included in the canine Nanostring Immune-Oncology (IO) panel used for the validation cohort. To summarize, the data from the validation cohort reinforces the unique gene findings in the discovery cohort.

Next, we validated expression of key genes at the protein level with IHC (Figure 3M). We found *CXCL10*, a gene associated with inflammatory responses, to be highly upregulated in many CID conditions including CCLE, MLE, and pyoderma (Supplementary Figure S2). We also found low levels of *IL17* expression, a gene involved in the immunopathogenesis of DLE (15), in CCLE (Supplementary Figure S3).

**Figure 4:**
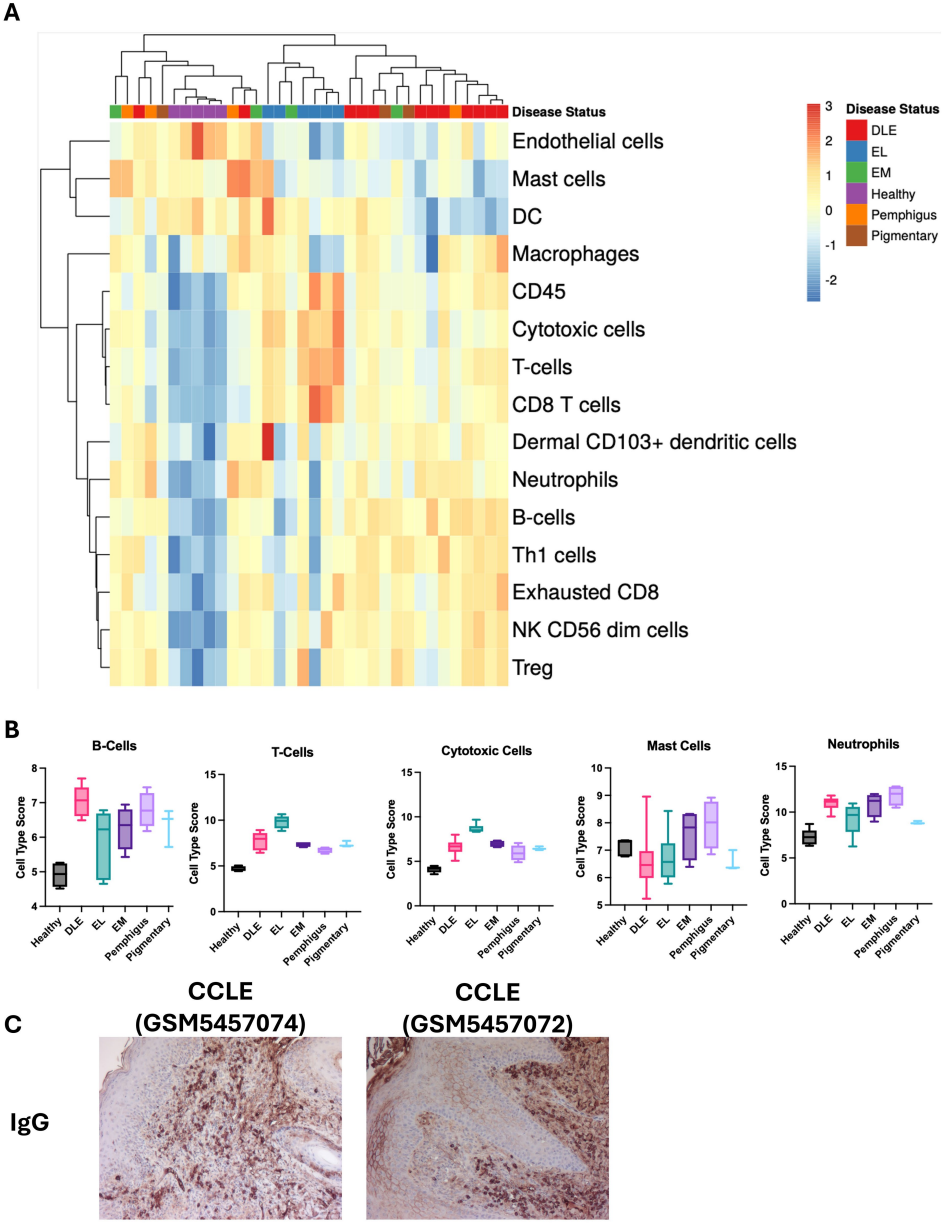
Examination of cell type signatures detected in different clinical subtypes of CID in dogs. **(A)** Heatmap of cell type profiling data from CID cases and healthy skin. **(B)** Graphs demonstrating enrichment of specific cell types in each condition (one-way ANOVAs with Tukey’s posttests ns). **(C)** Example canine IgG staining on histology sections from CCLE confirms the presence of B cells/plasma cells (10× magnification).

Last, we used hierarchical clustering to again organize by disease status (Figure 4A). Advanced Cell Type Analysis confidently predicted an increase in the number of B cells, T cells, Cytotoxic cells, Mast cells, and Neutrophils (Figure 4B). Of these, DLE had a significant increase in B cells (*p* < 0.0001), T cells (*p* < 0.0001), and cytotoxic cells (*p* < 0.0001). EL had a significant increase in T cells (*p* < 0.0001) and cytotoxic cells (*p* < 0.0001). Pemphigus had a significant increase in neutrophils (*p* < 0.0001). We performed IHC using canine IgG antibody to detect the presence of B cells/plasma cells and secreted antibodies in tissues and found strongest staining in CCLE (Figure 4C). Taken together, the data shows the upregulation of specific genes in a patient is correlated with an increase in infiltration of corresponding immune cell types, either by predicted chemokine-mediated recruitment or due to the presence of the cells themselves (i.e., cell-specific transcripts).

## Discussion

To avoid a trial-and-error period in treatment responses for CID patients, attacking specific molecular signatures may prove to be a more beneficial treatment plan. For patients diagnosed with DLE, *IFNG*, which was found in this study to be upregulated in this disorder, has been implicated in the pathogenesis of cytotoxic interface dermatitis and has been shown to increase keratinocyte sensitivity to cell mediated cytotoxicity in inflammatory skin conditions through JAK/STAT signaling (16). *IFNG* is uniquely and highly upregulated in human DLE as compared to other forms of CLE (17). Inhibiting *IFNG* using the monoclonal antibody AMG 811 improved gene signature scores in human trials of DLE (18). Harvey et al. (19) recently demonstrated that the JAK inhibitor oclacitinib is effective for CLE in dogs. It would also be interesting to test whether a caninized *IFNG* antibody would be efficacious for DLE in dogs, given the half-life of biologics tends to be longer.

For patients diagnosed with EL, *TRAT1*, a T cell-specific gene, could be a promising treatment avenue as this gene was found to be uniquely expressed and significantly upregulated in this disorder, which makes sense given the tumor is T cell-derived (11). *FOXO3A*, a gene known to possess tumor suppressor properties (20), was found to be significantly downregulated in EL. Currently, treatment avenues of EL patients besides chemotherapeutic agents include oclacitinib, glucocorticoids, and retinoids like Isotretinoin for countries where it is approved for veterinary use (21). However, pivoting disease management in EL toward upregulating *FOXO3A* could potentially serve to better control this disorder. Such medications could include metformin which, although considered to be a metabolic drug, could treat EL given its ability to stimulate AMPK-dependent expression of *FOXO3* (22). In mice, a study found that small doses of metformin selectively kill cancer cells (23). For EL patients, the effects of combining metformin with chemotherapy may vary depending on the type of chemotherapy. With chemoradiotherapy, treatment results and toxic side effects worsened in human patients taking metformin (24). Meanwhile, with adriamycin-cyclophosphamide plus paclitaxel (AC-T) chemotherapy, metformin reduced toxic effects such as oral mucositis in human patients (25). Given these varying results, further testing on the effects of metformin on chemotherapy patients should be investigated to safely administer this drug to EL patients.

We also found the potential for *CXCL8* to be a used for a targeted treatment plan given its unique expression in pemphigus patients in our meta-analysis. *CXCL8* has previously been reported by us and others to be elevated in pemphigus versus healthy control skin (12, 26). In this study, we also found a high neutrophil presence in pemphigus affected canines. CXCL8 is a known chemoattractant of neutrophils in inflammation that activates the G protein-coupled receptors CXCR1 and CXCR2 (27). Additionally, the other DEG we identified, *CSF3R*, is crucial for the formation of neutrophils through granulocyte progenitor maturation (28). Currently topical and systemic corticosteroids like glucocorticoids with or without azathioprine (29), or steroid-sparing agents besides azathioprine such as cyclosporine (30), oclacitinib (31), and mycophenolate mofetil (32) are used to treat canine pemphigus foliaceus and vulgaris (33). Based on our data, it may prove to be more effective to turn to other medications like ladarixin, which targets the interleukin-8 receptors *CXCR1* and *CXCR2*, or other small molecule inhibitors (34), to reduce the recruitment of neutrophils and reverse inflammatory processes (35–37). Furthermore, treatments like B-raf inhibitors that inhibit MAPK, a pathway vital to CSF3R upregulation, can be investigated and applied to pemphigus patients (38).

Additionally, *PECAM1*, a cell adhesion molecule, was found to be significantly downregulated in all CID disorders in this meta-analysis. This gene helps to maintain and restore the integrity of endothelial cell junctions after inflammation, a process contributing to vascular homeostasis (39). Low levels of *PECAM1* could increase vascular permeability, allowing numerous inflammatory cytokines to enter a patient’s vascular system (40), increasing the risk of vascular failure. Therefore, by targeting specific genes such as *IFNG*, *TRAT1* and *CXCL8*, this may help to control inflammation within these CID disorders.

Limitations of our study include small sample sizes, limited gene expression panels as compared to omics approaches and retrospective sample analyses. We were unable to identify unique gene expression in EM, likely due to the size of our panels, the heterogeneity of etiology, and differences in disease severity. Future studies could perform prospective collections and veterinary clinical trials testing the drugs we have discussed for the indicated CID conditions. If successful, these drugs could also be translated to human clinical trials.

To conclude, we identified shared and unique genes expressed in different CID conditions in client-owned dogs. We also identified recruitment of B-cells, T-cells, cytotoxic cells, and neutrophils suggesting differences in cells infiltrating in each of the separate CID conditions, contributing to the clinical inflammation patterns of CID patients. Validating *IFNG*, *TRAT1*, *CXCL8*, and *CSF3R* in a larger cohort would further reinforce the unique genes discovered, allowing them to be used for the development of a diagnostic tool and targeted treatment options in canines. Future implications include providing and enabling clinicians to use targeted treatment using these gene signatures in combination with current clinical approaches for CIDs.

## Materials and methods

### Study design

The main aim of this study was to discover unique gene signatures in different clinical presentations of CID using RNA isolated from diagnostic archival tissue biopsies.

### Ethics approval and consent to participate

At the time the veterinary patients were observed, samples were deposited with written owner consent in the Colorado State University Veterinary Diagnostic Laboratory or the Tufts Cummings School of Veterinary Medicine biorepository.

### Clinical samples

Samples were obtained as part of routine medical care under the guidance of a veterinarian at the Foster Hospital for Small Animals at Cummings School of Veterinary Medicine, spanning the years 2011–2019. Skin biopsies from the biorepositories were selected based on pathology reports. H&E sections were reexamined by a board-certified veterinary pathologist to confirm diagnoses and absence of obvious infectious disease, and clinical notes were reexamined by a board-certified veterinary dermatologist. Healthy control skin samples were obtained from leg margin biopsies from amputations. For the validation cohort, eight EL samples were obtained from the Colorado State University Veterinary Diagnostic Laboratory, seven of which yielded enough RNA for downstream analyses. Cases were reviewed by a board-certified veterinary pathologist to confirm diagnosis.

### Isolation of RNA from FFPE blocks

Thirty micrometers curls were cut from FFPE blocks. Using the Qiagen FFPE RNeasy kit, RNA was isolated per the manufacturer directions. RNase Zap was used to treat razor blades, excess paraffin was removed, and sections were incubated with deparaffinization solution (Qiagen). RNA was isolated using RNEasy FFPE kits per the manufacturer protocol and samples were quantified using a Nanodrop spectrophotometer (Fisher Scientific).

### Nanostring cartridge and processing

A custom designed Nanostring canine gene panel of 160 genes including cytokine, chemokine, and immune genes, as well as skin and immune cell specific transcripts was created as previously described (10). We used *B2M*, *RPL13A*, *CCZ1* and *HPRT* as housekeeping genes for this study. For the validation cohort, the NanoString canine Immune-Oncology (IO) panel was used. RNA (150 ng/assay) was hybridized for 18 h using a Bio-Rad C1000 touch thermal cycler, and samples were loaded into Nanostring cartridges and analyzed with a Sprint machine according to manufacturer’s instructions. New gene expression data are deposited on GEO under Accession # GSE213087 and GSE268931.

### Data analysis and statistics

nSolver, NanoString’s software, was used for all NanoString analysis, and GraphPad Prism was used to plot raw counts. Advanced analysis was used for the “cell Type Score,” a summary statistic of the expression of the marker genes for individual cell types found for each set of marker genes by taking its geometric mean of the log2-transformed normalized counts. These cell Type Scores were validated by NanoString against FACS and IHC (41). Heatmaps were generated using ClustVis software (42) and Venn diagrams were generated using BioVenn software (14). Normalized counts were graphed and analyzed using GraphPad Prism. Normality tests were performed to determine which tests were most appropriate, and One-way ANOVAs were used to examine normally distributed data, and the Kruskal–Wallace tests were used to examine non-normally distributed data, with *post hoc* tests to examine *p*-value differences between groups. A statistically significant difference was considered as *p* < 0.05.

### Immunohistochemistry (IHC)

The IHC was performed on 5 μm sections using anti-IFN-*γ* (catalog # 363576; US Biological), anti-IL17 (catalog # 140996; US Biological), anti-CXCL8 (catalog # 141074; US Biological), anti-CXCL10 (catalog # 140923; US Biological), anti-Foxo3a (catalog # F9049-30Q; US Biological), anti-canine IgG (catalog # 7316; Novus Biologicals) or isotype control (Biolegend catalog # 910801) at 1:100 dilution using a Dako automated slide staining machine. All sections were counterstained with hematoxylin. H&E images were taken using an Olympus BX51 microscope with Nikon NIS Elements software version 3.10, and IHC images were taken using an Olympus BX40 microscope with cellSens Entry software version 1.14 and a Zeiss Axio Imager M1 with AmScope software.

## Data Availability

The datasets presented in this study can be found in online repositories. The names of the repository/repositories and accession number(s) can be found in the article/Supplementary material.
