# Supplementary material for: Evaluating biomarkers in canine cytotoxic interface dermatitis reactions to account for clinical and histopathological similarities and differences
Source: Front Vet Sci. 2025 Jan 22;11:1471590. doi: 10.3389/fvets.2024.1471590 (PMC11796617; doi:10.3389/fvets.2024.1471590)
Supplement: Supplementary file 1 [file Supplementary_file_1.docx]

**
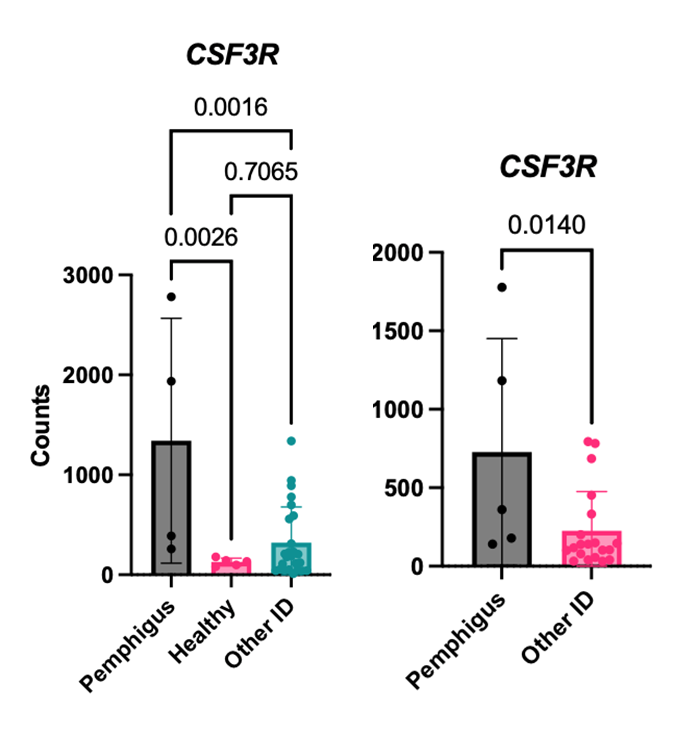
**

**Figure S1.** Significance of *CSF3R* in the discovery and validation cohorts (one-way ANOVAs with Tukey's posttests significant as indicated).
