# Supplementary material for: Evaluating biomarkers in canine cytotoxic interface dermatitis reactions to account for clinical and histopathological similarities and differences
Source: Front Vet Sci. 2025 Jan 22;11:1471590. doi: 10.3389/fvets.2024.1471590 (PMC11796617; doi:10.3389/fvets.2024.1471590)
Supplement: Supplementary file 2 [file Supplementary_file_2.docx]

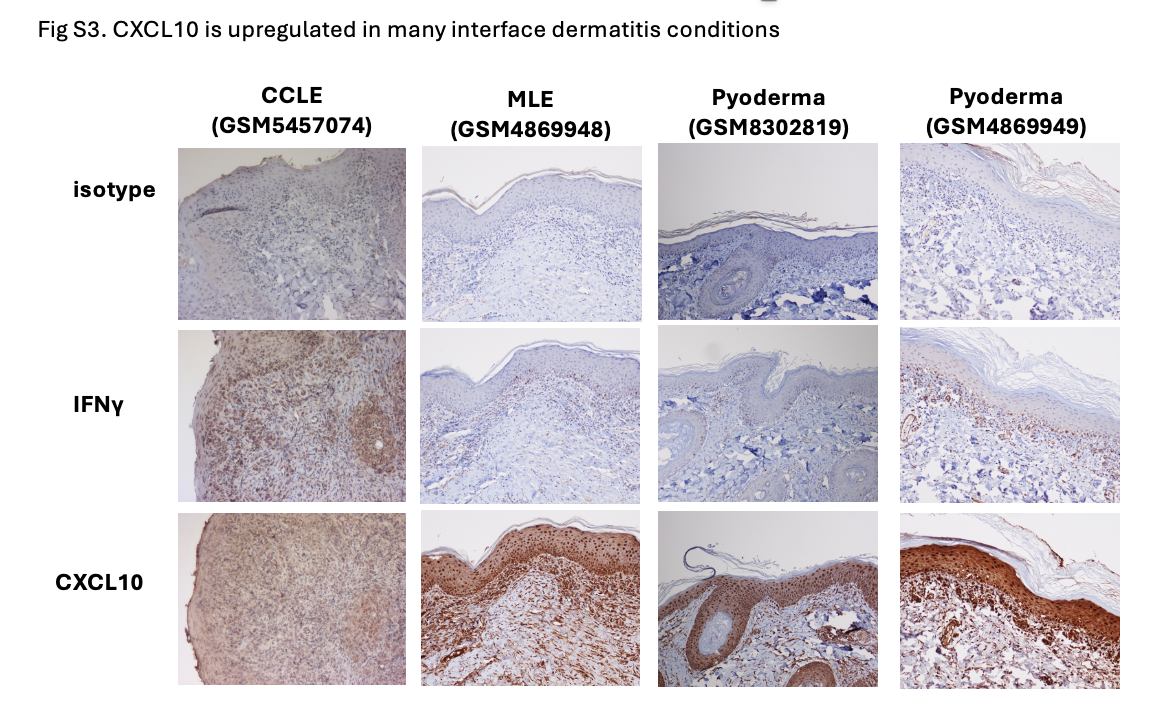


**Figure S2.** Isotype, IFN-γ, and CXCL10 staining in histology sections from CCLE, MLE, and pyoderma. CXCL10 is upregulated in the epidermis of many CID conditions, whereas IFN-γ is more uniquely expressed in DLE.
