# Supplementary material for: Evaluating biomarkers in canine cytotoxic interface dermatitis reactions to account for clinical and histopathological similarities and differences
Source: Front Vet Sci. 2025 Jan 22;11:1471590. doi: 10.3389/fvets.2024.1471590 (PMC11796617; doi:10.3389/fvets.2024.1471590)
Supplement: Supplementary file 3 [file Supplementary_file_3.docx]

*
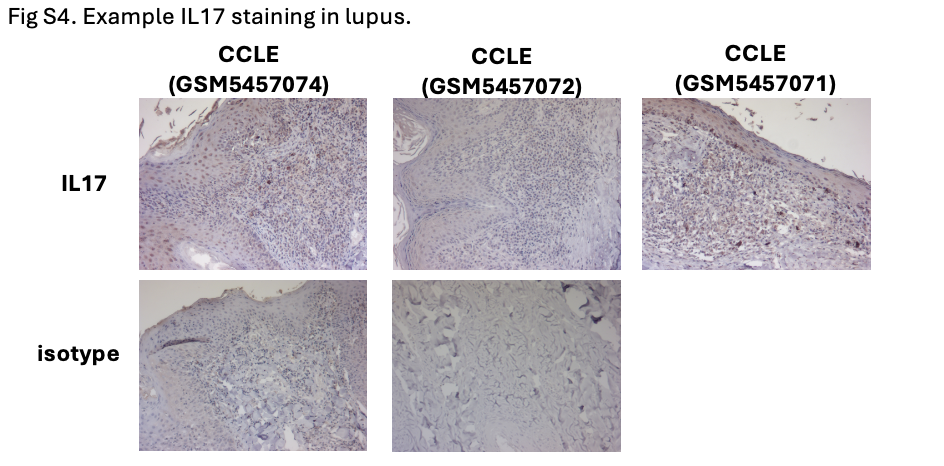
*

**Figure S3.** IL17 and isotype staining in histology sections from CCLE demonstrating low levels of IL17 staining, evaluated using subjective qualitative analysis.
